# FGF21 mediates alcohol-induced adipose tissue lipolysis by activation of systemic release of catecholamine in mice[S]

**DOI:** 10.1194/jlr.M058610

**Published:** 2015-08

**Authors:** Cuiqing Zhao, Yanlong Liu, Jian Xiao, Liming Liu, Shaoyu Chen, Moosa Mohammadi, Craig J. McClain, Xiaokun Li, Wenke Feng

**Affiliations:** * College of Basic Medical Sciences, Jilin University, Changchun, China; † Departments of Medicine University of Louisville School of Medicine, Louisville, KY; § Pharmacology and Toxicology, University of Louisville School of Medicine, Louisville, KY; ** School of Pharmaceutical Sciences, Wenzhou Medical University, Wenzhou, China; †† Department of Pharmacology, New York University School of Medicine, New York, NY; §§ Robley Rex Veterans Administration Medical Center, Louisville, KY

**Keywords:** adipose tissue, lipolysis and fatty acid metabolism, liver, cell signaling, alcoholic liver disease, fibroblast growth factor 21

## Abstract

Alcohol consumption leads to adipose tissue lipoatrophy and mobilization of FFAs, which contributes to hepatic fat accumulation in alcoholic liver disease. This study aimed to investigate the role of fibroblast growth factor (FGF)21, a metabolic regulator, in the regulation of chronic-binge alcohol-induced adipose tissue lipolysis. FGF21 KO mice were subjected to chronic-binge alcohol exposure, and epididymal white adipose tissue lipolysis and liver steatosis were investigated. Alcohol exposure caused adipose intracellular cAMP elevation and activation of lipolytic enzymes, leading to FFA mobilization in both WT and FGF21 KO mice. However, alcohol-induced systemic elevation of catecholamine, which is known to be a major player in adipose lipolysis by binding to the β-adrenergic receptor, was markedly inhibited in KO mice. Supplementation with recombinant human FGF21 to alcohol-exposed FGF21 KO mice resulted in an increase in fat loss in parallel with an increase of circulating norepinephrine concentration. Furthermore, alcohol consumption-induced fatty liver was blunted in the KO mice, indicating an inhibition of fatty acid reverse transport from adipose to the liver in the KO mice. Taken together, our studies demonstrate that FGF21 KO mice are protected from alcohol-induced adipose tissue excess-lipolysis through a mechanism involving systemic catecholamine release.

Adipose tissue is a specialized connective tissue that functions as the major storage site for fat in the form of TGs. Serving as an energy reservoir, adipose tissue synthesizes TGs when energy intake exceeds energy output. During fasting or in response to stress, adipose tissue mobilizes FFAs and glycerol (lipolysis), providing other tissues with metabolites and energy substrates (1, 2). While lipolysis is a physiological response to metabolic changes, excess lipolysis may lead to increased circulating FFA levels, which is a risk factor for insulin resistance and fatty liver. Clinical and experimental animal studies showed that white adipose tissue (WAT) hyperlipolysis was associated with increased hepatic fat accumulation in alcoholic liver disease (ALD) (3, 4). Reducing lipolysis by dietary supplementation decreased fatty liver in mice with ALD (5, 6).

Although alcohol-associated adipose tissue lipolysis contributes to the development and progression of ALD in patients and in animal models, the underlying mechanisms are not yet clear. Fibroblast growth factor (FGF)21 is an FGF family member produced by the liver and other metabolic tissues that plays an important role in energy homeostasis and glucose and lipid metabolism (7). Systemic administration of FGF21 alters lipid profiles in animal models (8, 9), in part, by regulating lipolysis in WAT. However, the exact action of FGF21 on WAT lipolysis remains elusive. The phenotypes of FGF21 transgenic mice suggest that FGF21 stimulates adipose tissue lipolysis (10), while other studies showed that FGF21 attenuates hormone-stimulated lipolysis in both human and murine adipocytes (11). A recent study suggests that the role of FGF21 in adipose tissue lipolysis is metabolic state dependent; FGF21 stimulates lipolysis in the WAT during normal feeding but inhibits it during fasting (12).

To elucidate the roles of FGF21 in alcohol-induced adipose tissue lipolysis, we exposed FGF21 KO mice to alcohol using a chronic-binge model. Absence of FGF21 attenuates alcohol-induced lipolysis in WAT, which is likely mediated by sympathetic nervous system (SNS) activation.

## MATERIALS AND METHODS

### Animal experiments

Male C57BL/6J mice (WT) and FGF21 KO mice (13) were used for this study. Alcohol-fed (AF) groups were allowed free access to the liquid diet (Lieber DeCarli; Research Diets, Inc., New Brunswick, NJ) containing 5% (w/v) alcohol for 12 days, and control groups were pair-fed (PF) with the isocaloric maltose dextrin (14) in the following groups: WT+PF, WT+AF, KO+PF, and KO+AF. The mice in the PF groups were given the same amount of food consumed by the mice in AF groups in the previous day. On the last day of the experiment, mice were gavaged with a single dose of alcohol (5 g/kg body weight) or isocaloric maltose dextrin. Mice were euthanized 6 h later. One group of alcohol-exposed FGF21 KO mice was treated with 4 mg/kg recombinant human FGF21 (rhFGF21) (KO+AF+rhFGF21) (15) via intraperitoneal injection during the last 5 days. rhFGF21 was produced in *Escherichia coli* and purified to be endotoxin free (16). The treatment schedule is illustrated in supplementary Fig. 1. At the end of the experiment, the mice were anesthetized with Avertin (2,2,2-tribromoethanol) and plasma and tissue samples were collected for assays. All mice were housed under controlled lighting (6:00 AM to 6:00 PM light cycle/6:00 PM to 6:00 AM dark cycle). All mice were treated according to the protocols reviewed and approved by the Institutional Animal Care and Use Committee of the University of Louisville.

### Statistical analysis

Two-way ANOVA with Bonferroni’s post hoc test, one-way ANOVA with Tukey’s post hoc test, or two-tailed unpaired Student’s *t*-test were used for the determination of statistical significance of the data where they were appropriate. All statistical analyses were performed with GraphPad Prism software version 5 (GraphPad Software, Inc., San Diego, CA). For animal studies, each experimental group had seven mice. For cell culture studies, experiments were repeated three times in triplicate for each experiment. Results are expressed as mean ± SEM. Differences between groups were considered significant at *P < 0.05, **P < 0.01, and ***P < 0.001.

Additional methods are described in the supplementary Materials and Methods.

## RESULTS

### Alcohol exposure increases FGF21 expression

**Fig. 1.**
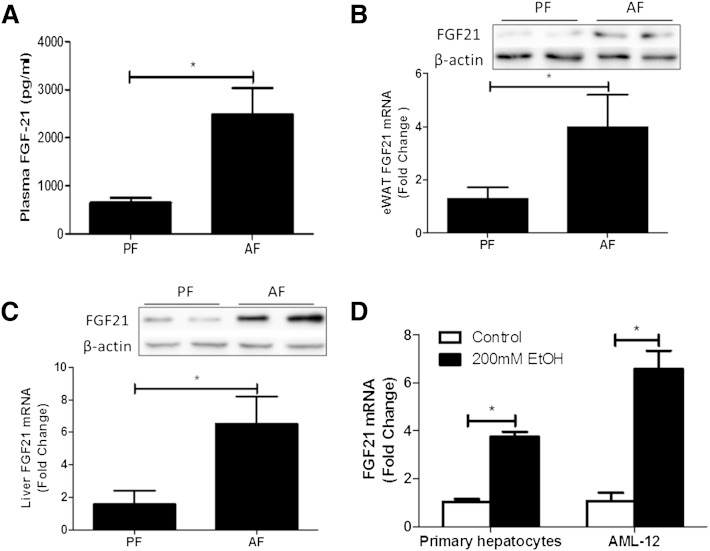
Effects of alcohol on FGF21 expression. C57BL/6 J mice were fed Lieber DeCarli liquid diet containing 5% alcohol (AF) or pair-fed isocaloric maltose dextrin diet (PF) as described in the Materials and Methods. On the last day of the experiment, AF and PF mice were gavaged with a single dose of alcohol (5 g/kg body weight) or isocaloric maltose dextrin, respectively, and euthanized 6 h later. A: Plasma FGF21 protein levels. B: FGF21 protein (top) and mRNA (bottom) levels in eWAT. C: FGF21 protein (top) and mRNA (bottom) levels in liver. D: mRNA levels of FGF21 in primary hepatocytes isolated from WT mice and in AML-12 cells after 200 mM ethanol treatment for 4 h.

The liver is considered the main source for the production of FGF21. Extrahepatic tissues, including white and brown adipose tissue, also express FGF21 (17). To determine whether alcohol exposure affects FGF21 expression, we exposed 8- to 10-week-old mice to alcohol in a chronic-binge exposure model as described in the Materials and Methods. Chronic-binge alcohol exposure increased plasma FGF21 concentration by 4-fold approximately (**Fig. 1A**). A marked increase in FGF21 gene expression and protein concentration in both liver and epididymal WAT (eWAT) was observed, as shown in Fig. 1B, C. Furthermore, to determine whether the hepatocyte responds to alcohol for FGF21 expression, we incubated mouse primary hepatocytes and AML-12 (a hepatocyte cell line) cells with 200 mM ethanol for 4 h (200 mM shown here. Positive dose-response starting at 50 mM, data not shown). FGF21 mRNA levels were increased nearly 4 and 7 times in primary hepatocytes and AML-12 cells, respectively, after alcohol exposure (Fig. 1D).

### FGF21 deficiency markedly reduces chronic-binge alcohol exposure-induced eWAT lipolysis

**Fig. 2.**
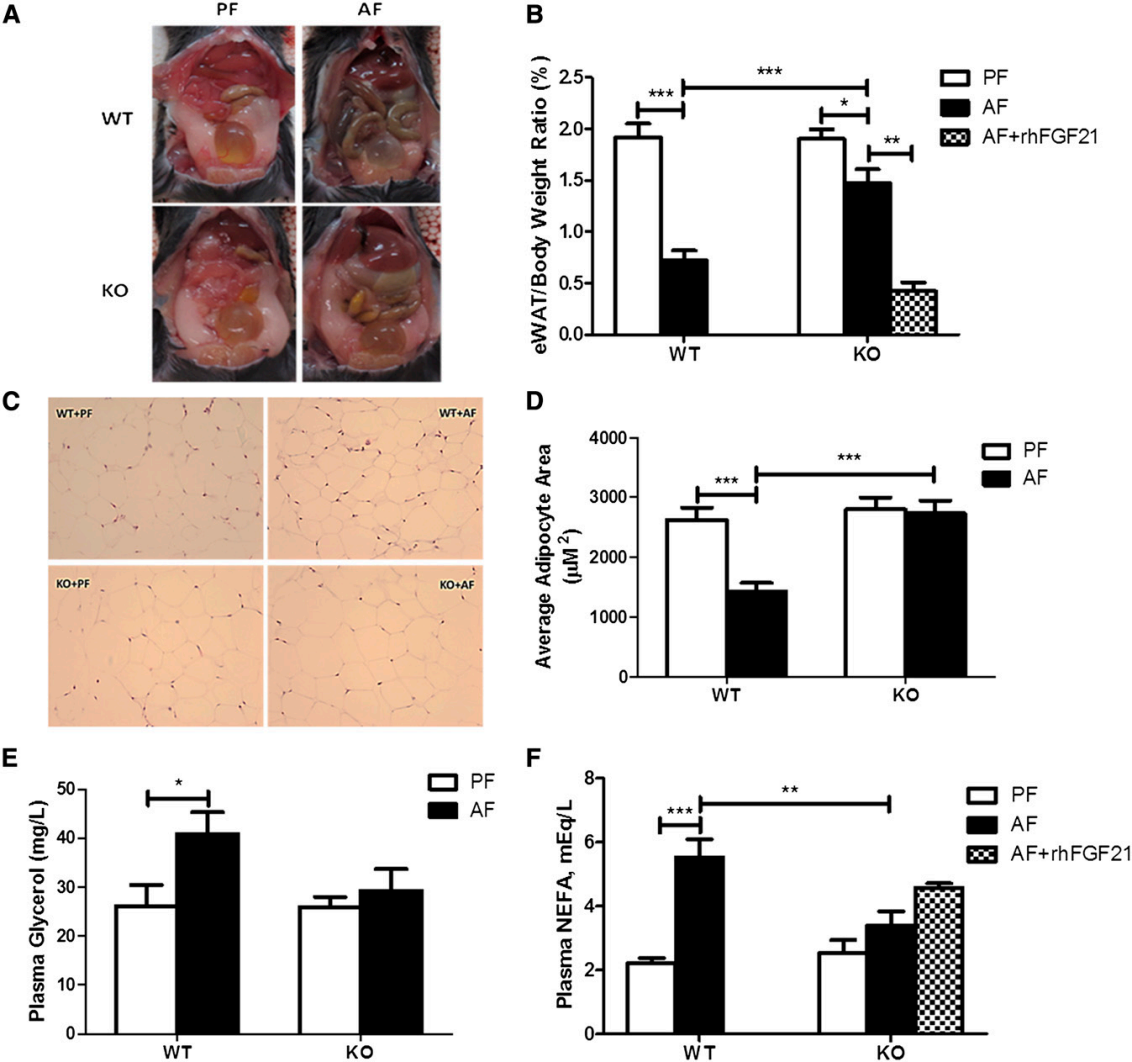
FGF21 deficiency markedly reduces chronic-binge alcohol exposure-induced eWAT lipolysis. FGF21 KO mice and their WT controls were treated as described in the Materials and Methods. The mice in the KO+AF group were injected intraperitoneally with rhFGF21 at a dose of 4 mg/kg body weight once a day for the last 5 days. A: Epididymal adipose tissue image. B: Epididymal adipose tissue/body weight ratio. C: Histopathology of eWAT depots (200×). D: Quantification of adipocyte size. E: Plasma glycerol concentrations. F: Plasma NEFA concentrations.

FGF21 KO mice have similar eWAT size compared with WT mice. Chronic-binge alcohol exposure markedly reduced eWAT weight in WT mice. Surprisingly, this eWAT weight loss was significantly reduced in FGF21 KO mice (**Fig. 2A**). The ratio of eWAT to total body weight was reduced approximately 62% in WT mice by alcohol exposure, but only a 22% reduction was observed in FGF21 KO mice (Fig. 2B). In addition, adipocyte size was reduced about 50% in WT mice, but unchanged in FGF21 KO mice, as measured by hematoxylin and eosin staining of eWAT (Fig. 2C, D). These observations indicate that FGF21 KO mice are resistant to alcohol-induced lipolysis in eWAT. To further characterize the role of FGF21 in alcohol-mediated adipose tissue lipolysis, we measured glycerol and NEFA levels in the circulation. Chronic-binge alcohol exposure significantly increased plasma glycerol and NEFA concentrations in WT mice, but not in FGF21 KO mice (Fig. 2E, F).

**Fig. 3.**
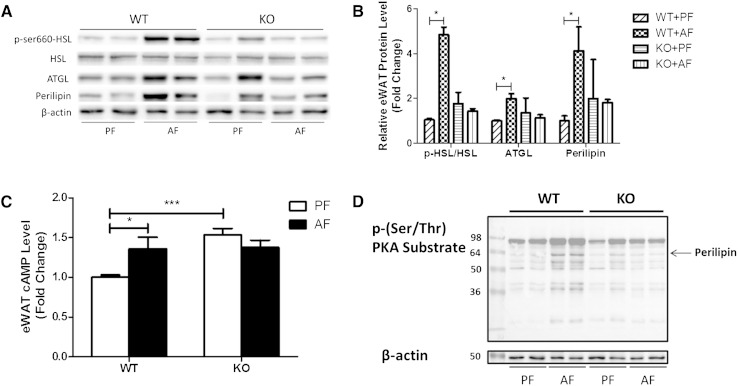
FGF21 KO mice have reduced expression and activity of proteins involved in adipose tissue lipolysis. Mice were fed as described in the Materials and Methods. Proteins in eWAT were analyzed by Western blotting. A: Levels of p-HSL, ATGL, and PLIN. B: The quantification of protein bands in (A) by densitometry analysis; β-actin levels served as loading controls. C: Epididymal adipose tissue cAMP concentrations. D: The p-(Ser/Thr) PKA substrate levels; β-actin levels as loading controls.

To further understand the role of FGF21 in adipose tissue, we analyzed the mRNA and protein levels, and the activation of a set of genes known to regulate lipolysis in eWAT. Alcohol exposure did not change the mRNA expression of hormone sensitive lipase (HSL), adipose tissue TG lipase (ATGL), and perilipin (PLIN) in either WT or FGF21 KO mice (data not shown), but markedly increased HSL-ser660 phosphorylation and ATGL and PLIN protein levels (**Fig. 3A, B**). It is known that HSL phosphorylation is regulated by protein kinase A (PKA) activation, which is mediated by adipose cAMP. eWAT cAMP levels were markedly increased in WT mice compared with KO mice in response to alcohol exposure (Fig. 3C). PLIN is a coating protein on the lipid droplets in adipocytes. PKA phosphorylates PLIN, exposing the lipid droplet to HSL-mediated lipolysis. We used anti-p-(Ser/Thr) PKA substrate antibody to detect adipose PKA substrates, including PLIN, which were significantly phosphorylated by alcohol exposure in the eWAT of WT mice, while they were inhibited in the KO mice (Fig. 3D). This approach has been used in previous studies to determine PKA-mediated PLIN phosphorylation (18).

**Fig. 4.**
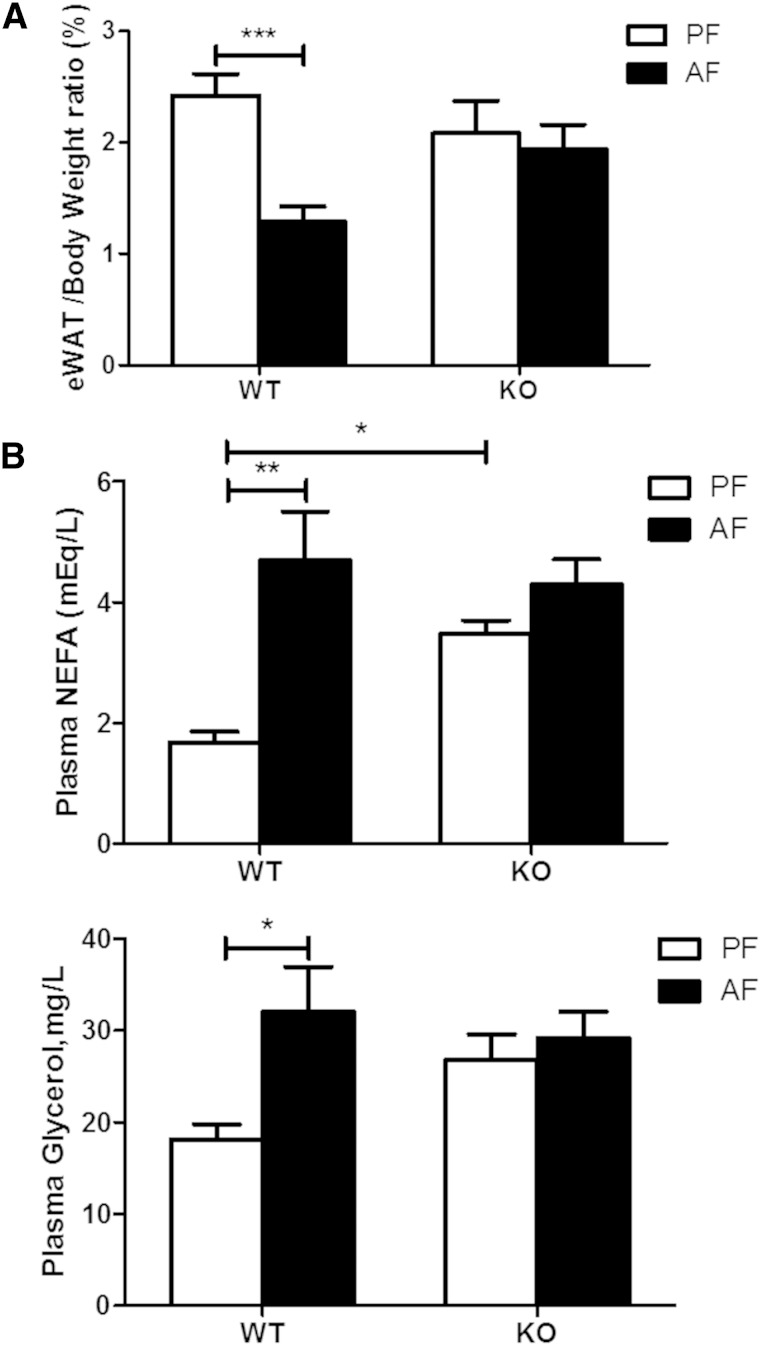
FGF21 KO mice display attenuated eWAT lipolysis by chronic or acute alcohol exposure. For chronic alcohol exposure, C57BL/6 J and FGF21 KO mice were fed Lieber DeCarli liquid diet containing 5% alcohol (AF) or pair-fed isocaloric maltose dextrin diet (PF) for 4 weeks. A: eWAT/body weight ratio. For acute alcohol exposure, C57BL/6 J mice and FGF21 KO mice were gavaged in the early morning with a single dose of alcohol (5 g/kg body weight (AF) or isocaloric maltose dextrin (PF). Blood was collected 6 h later. B: Plasma NEFA and glycerol concentrations.

The effects of alcohol exposure models were also examined. Chronic alcohol consumption (4 weeks) markedly reduced the eWAT/body weight ratio in WT mice, but not in the KO mice (**Fig. 4A**). Similarly, acute alcohol exposure (one gavage of 5 g/kg alcohol) induced changes in circulating glycerol, and NEFAs were more pronounced in the WT mice than in the KO mice (Fig. 4B). Thus, FGF21 KO mice are consistently resistant to adipose lipolysis in three different alcohol-exposure models. In the following studies, we focused on the chronic-binge alcohol exposure model.

### Insulin signaling is not associated with alcohol-induced adipose lipolysis in FGF21 KO mice

**Fig. 5.**
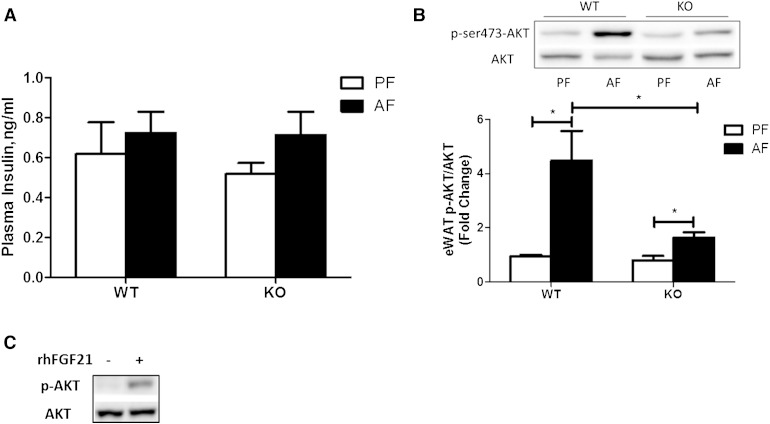
Insulin is not correlated with alcohol-induced adipose dysfunction. Mice were fed as described in the Materials and Methods. A: Plasma insulin levels. B: Immunoblot analysis of eWAT phospho-AKT ser-473, AKT protein levels (top), quantification of the immunoblot bands (bottom). C: 3T3-L1 adipocytes were treated with vehicle or 1 μg/ml rhFGF21 for 5 min. Cell lysates were immunoblotted for phospho-AKT ser-473 or total AKT as indicated.

Insulin is well-known as a major antilipolytic hormone acting to limit release of fatty acids from adipose tissue, and insulin signaling pathways are also targets of alcohol (19). To determine the role of insulin in the regulation of lipolysis by FGF21 in response to alcohol exposure, we analyzed insulin and Akt activation. Plasma insulin levels did not differ between mouse groups of PF and AF in WT and FGF21 KO mice (**Fig. 5A**). Alcohol exposure increased Akt phosphorylation, which is known to be a downstream target of insulin, in both WT and FGF21 KO mice (Fig. 5B). To determine whether FGF21 directly activates Akt signaling, we incubated fully differentiated 3T3-L1 adipocytes with rhFGF21. Western blot analysis showed that FGF21, indeed, increased phosphorylation levels of Akt in adipocytes (Fig. 5C). Due to the antilipolytic effect of insulin, the increased insulin signaling would attenuate alcohol-induced adipose tissue lipolysis. As mentioned above, however, alcohol exposure increased eWAT lipolysis, indicating that insulin signaling is unlikely to be the major causative factor in alcohol-induced lipolysis.

### The adipocyte does not respond to FGF21 and alcohol to induce lipolysis

To further investigate the mechanisms of FGF21 and alcohol-mediated lipolysis in adipocytes, we measured the median glycerol concentration in fully differentiated 3T3-L1 cells and primary mouse adipocytes exposed to alcohol or rhFGF21. As shown in supplementary Fig. 2A, differentiated 3T3-L1 cells responded to rhFGF21, as evidenced by the increased phosphorylation of ERK. Isoproterenol, a synthetic catecholamine, significantly increased median glycerol concentrations in both primary adipocytes and fully differentiated 3T3-L1 adipocytes. A sympatholytic nonselective β-blocker (propranolol) inhibited isoproterenol-induced lipolysis in primary adipocytes. However, alcohol and rhFGF21 had no lipolytic effect in either cell type (supplementary Fig. 2B, C), indicating that neither alcohol nor FGF21 directly acts on adipocytes to induce lipolysis. The effects of alcohol, rhFGF21, and isoproterenol on glycerol release were similar in the primary adipocytes isolated from WT and FGF21 KO mice (supplementary Fig. 2B), further suggesting that local action of FGF21 does not affect adipose lipolysis.

### FGF21 mediates alcohol-enhanced catecholamine release

**Fig. 6.**
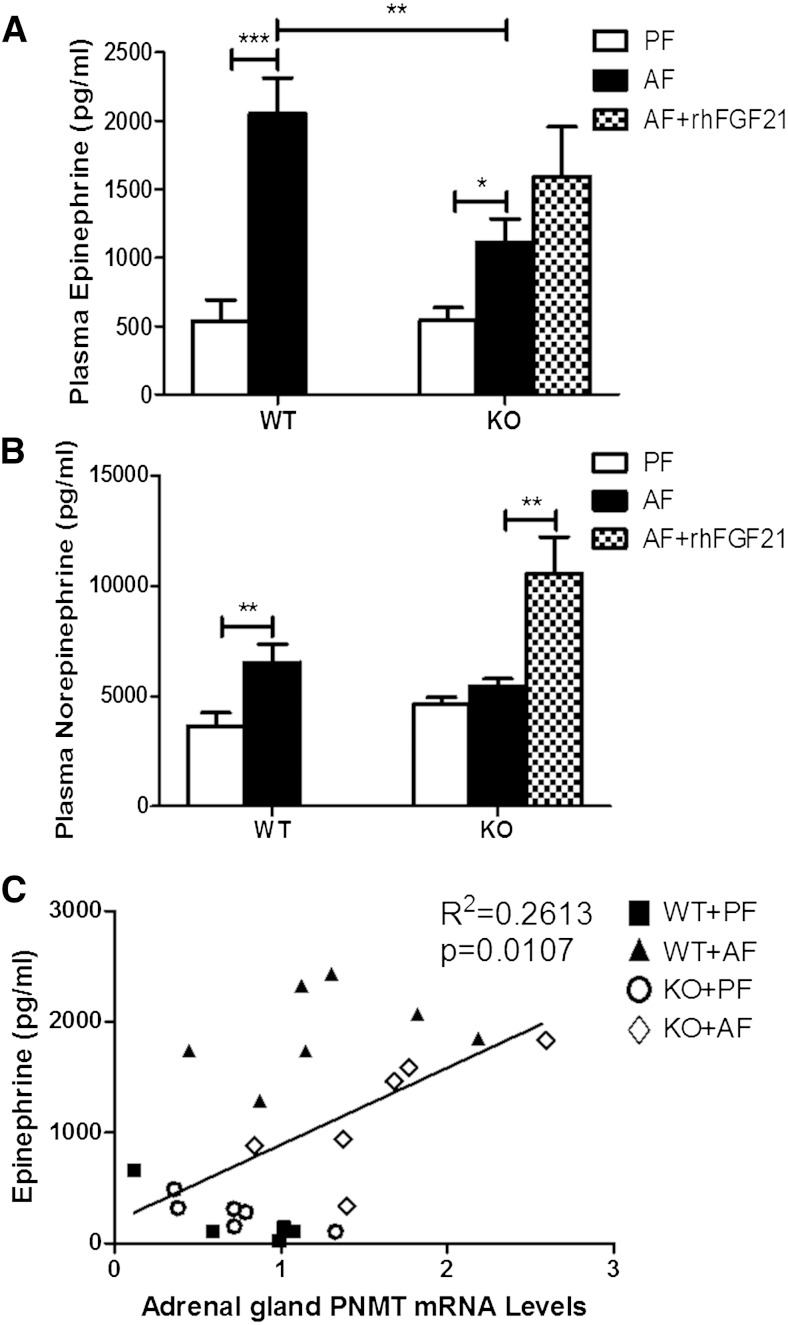
FGF21 KO mice have reduced plasma levels of EP and NE in response to alcohol exposure. Mice were fed as described in the Materials and Methods, the mice in KO+AF group were injected intraperitoneally with rhFGF21 at a dose of 4 mg/kg body weight once a day for the last 5 days. A: Plasma EP levels. B: Plasma NE levels. C: Linear correlation of the adrenal gland PNMT mRNA expression and plasma EP level.

Next, we investigated the effects of alcohol exposure on catecholamine release. Epinephrine (EP) and norepinephrine (NE), secreted through SNS innervation, have been acknowledged as the principal initiators of lipolysis (20–22). To explore the mechanisms by which alcohol stimulates lipolysis, plasma EP and NE were measured. Alcohol exposure lead to about a 4-fold increase in EP release (**Fig. 6A**), and about a 2-fold increase in NE release (Fig. 6B) in WT mice. However, in FGF21-deficient mice, alcohol induced only about a 2-fold increase in EP release and virtually no change for NE (Fig. 6A, B). The plasma EP concentrations were positively correlated with the mRNA levels of phenylethanolamine *N*- methyltransferase (PNMT), which is a major enzyme involved in EP synthesis in the adrenal gland (Fig. 6C). As PNMT activity is largely regulated by adrenal glucocorticoids, we measured plasma corticosterone levels. Alcohol exposure significantly increased plasma corticosterone levels in both WT and KO mice. However, the elevation in the KO mice was less significant than in WT mice (data not shown). EP and NE stimulate lipolysis through promoting β-adrenergic receptor (β-AR) activation (23, 24). β-ARs have three subtypes, β_1_-, β_2_-, and β_3_-AR. As shown in supplementary Fig. 3A, β_3_-AR had the highest expression level in eWAT (^Δ^Ct values). However, no difference was found in chronic-binge alcohol exposure-induced β-AR expression between eWAT in WT and KO mice (supplementary Fig. 3B–D).

### Exogenous FGF21 administration exacerbates chronic-binge alcohol exposure-induced lipolysis

Based on the above findings, we hypothesized that FGF21 may enhance chronic-binge alcohol exposure-induced eWAT lipolysis through the regulation of systemic catecholamine release. To test this hypothesis, one group of alcohol-exposed FGF21 KO mice (KO+AF+FGF21) was treated with 4 mg/kg/day rhFGF21 via intraperitoneal injection for the last 5 days. As expected, rhFGF21 treatment significantly increased eWAT weight loss. Compared with the alcohol exposure group, the ratio of eWAT to total body weight was decreased 71% after rhFGF21 treatment in KO mice (Fig. 2B). Interestingly, the decrease in eWAT by rhFGF21 administration was accompanied by an increase in EP and NE in the plasma (Fig. 6A, B). The increased lipolysis in the KO mice by rhFGF21 was further supported by the increased plasma NEFA levels (Fig. 2F), although the change did not reach statistical significance.

### Effects of FGF21 deletion on chronic-binge alcohol-induced hepatic steatosis and injury

**Fig. 7.**
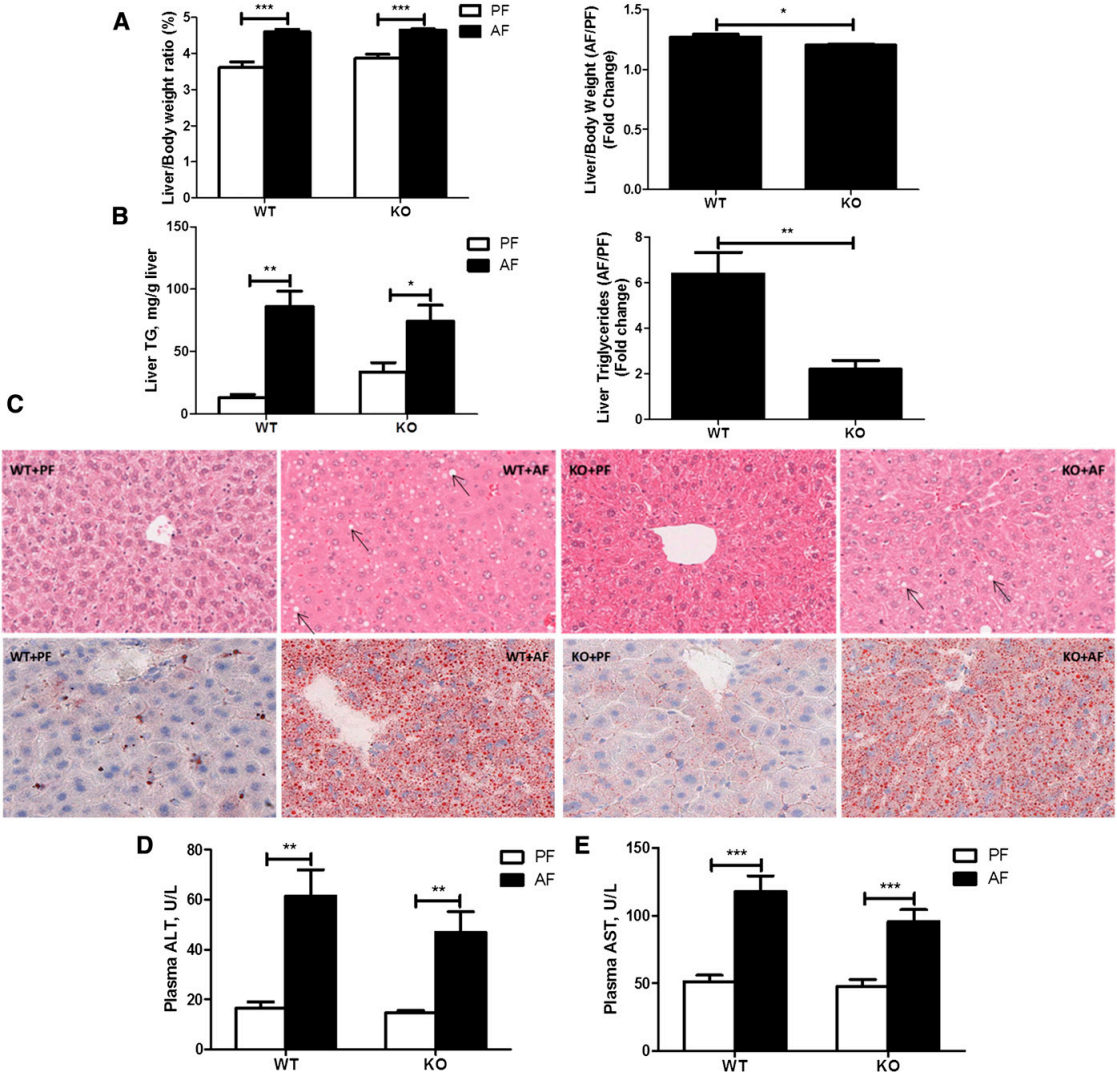
Effects of FGF21 deletion on chronic-binge alcohol-induced hepatic steatosis and injury. Mice were fed as described in the Materials and Methods. A: Liver to body weight ratios. B: Liver TG concentrations. C: Hematoxylin and eosin (upper) and Oil Red O (below) staining of hepatic tissues (arrows indicate lipid droplets). D: Plasma ALT concentrations. E: Plasma AST concentrations.

Adipose tissue lipolysis contributes to chronic-alcohol-induced hepatic steatosis (4). To determine whether FGF21-mediated adipose lipolysis is involved in the chronic-binge alcohol exposure-induced fatty liver, the hepatic steatosis index and liver markers of injury were measured. Alcohol exposure significantly increased liver/body weight ratios in both WT and FGF21 KO mice, and the change in FGF21 KO mice was smaller than in WT mice (**Fig. 7A**). Similarly, there were significant changes in liver TG concentrations by alcohol exposure in both WT and KO mice (Fig. 7B). Notably, chronic-binge alcohol exposure increased liver TG levels by about 6-fold in the WT mice, but only by about 2-fold in the KO mice. These results indicate that the KO mice are less sensitive to chronic-binge alcohol exposure in hepatic fat accumulation (Fig. 7B). A baseline increase in TG levels in the KO+PF mice may contribute to the reduced sensitivity to chronic-binge alcohol exposure, but insignificantly. Confirming the biochemical assays, the histological examinations showed a reduced fat accumulation in the livers of the KO mice in response to alcohol exposure (Fig. 7C). Alcohol exposure significantly increased plasma alanine aminotransferase (ALT) and aspartate aminotransferase (AST) levels (Fig. 7D, E) in WT mice, and these elevations tended to be reduced in the KO mice, indicating a likely reduction of liver injury by chronic-binge alcohol exposure in the KO mice.

## DISCUSSION

Alcohol-induced hepatic fat accumulation is considered to be the earliest pathological alteration in ALD. Clinical and experimental studies have demonstrated that a reduction of in situ liver lipogenesis and an increase of lipid β-oxidation prevent hepatic steatosis and slow or halt the progression of ALD (25, 26). On the other hand, alcohol-induced fatty liver disease is associated with reverse transport of FFAs derived from adipose lipoatrophy by alcohol ingestion. The mechanisms by which alcohol disrupts adipose tissue homeostasis to contribute to alcoholic fatty liver disease are not fully understood. In the current study, we demonstrated that chronic-binge alcohol exposure significantly reduced epididymal adipose fat mass, but this reduction was inhibited in the FGF21 KO mice. Supplementation of the rhFGF21 to the FGF21 KO mice further exacerbated alcohol-induced adipose lipolysis. Importantly, we showed that the function of FGF21 was associated with elevation of circulating catecholamine concentrations due to alcohol exposure.

Adipose tissue lipolysis is an exquisitely controlled process (27, 28). cAMP signaling represents the principal prolipolytic pathway in WAT. cAMP activation stimulates PKA activation, which mediates the activation of HSL by phosphorylation. PLIN is a coating protein that binds to the surface of lipid droplets and appears to be essential for lipid degradation (29). PKA activation promotes HSL phosphorylation at Ser 660, which is crucial for its activation and translocation to PLIN-containing droplets (30, 31), where HSL catalyzes the hydrolysis of diglycerides to monoglycerides (32, 33). Recently, another lipase, ATGL, has been identified. ATGL favors TG substrates and is a rate-determining enzyme for lipolysis in adipose tissue (34, 35). ATGL is not a direct substrate for PKA (35) and its activity depends on PLIN activation (36). These concepts were supported by our current study, which showed that the levels of ATGL, PLIN, and p-HSL were increased in the eWAT by chronic-binge alcohol exposure.

Adipose lipid metabolism is known to be affected by hormones (37). Lipolysis is negatively regulated by insulin and positively regulated by catecholamine (38). Insulin exerts its influence by stimulating the phosphoinositide 3-kinase-Akt pathway in the adipose tissue. Earlier studies showed that insulin-induced tyrosine phosphorylation of phosphoinositide 3-kinase and phosphorylation of Akt were affected by chronic alcohol feeding (3, 39), leaving the effect of alcohol on insulin signaling in adipose tissue elusive. It is, however, an important issue because cross-talk between insulin and FGF21 has been demonstrated (40–42). Exogenous FGF21 administration increased insulin sensitivity in several animal models of metabolic diseases (8, 43, 44). We showed that plasma insulin levels were not affected, but phosphorylation of Akt was increased by alcohol exposure, indicating an activation of insulin action. Our observations are different than a previous study in which an inhibition of insulin action by chronic alcohol exposure was demonstrated (4). The differences may be due to the various alcohol exposure models. In fact, the chronic and binge alcohol exposure models produced remarkable differences in the reverse transport of FFAs derived from adipose lipolysis into the liver in animal models (45, 46). Nevertheless, adipose lipolysis is increased by both chronic and acute alcohol exposure. Thus, the potentially increased insulin action observed in chronic-binge alcohol groups might inhibit lipolysis, but this inhibition may be overridden by other stimulatory factors.

A major extra-adipose signaling pathway for adipose lipolysis is catecholamine signaling. Catecholamines (EP and NE) (47), the end mediators of the sympatho-adrenergic system, are secreted mainly from the adrenal medulla by SNS activation and have been implicated as important modulators of lipolytic activity. Catecholamines are known to act physiologically via binding to β-ARs (subtypes β_1–3_-ARs). β_1_-AR is found predominantly in the brain and heart (48), β_2_-AR is widely expressed (49), and β_3_-AR is mainly expressed in adipose tissue (50). Binding to adipose tissue β-ARs, catecholamines activate Gs protein and enhance intracellular cAMP concentration and stimulate cAMP-dependent PKA activation, leading to the phosphorylation and activation of HSL (51). Chronic-binge alcohol exposure does not act directly on the adipose tissue to stimulate lipolysis. Instead, we showed that alcohol exposure increased SNS activity and stimulated the release of NE and EP in the circulation to activate β-AR. This agrees with the clinical observation that alcohol ingestion elevates blood catecholamine levels (52).

The most important finding in the current study is that FGF21 mediates catecholamine release in response to alcohol exposure. We showed that chronic-binge alcohol-induced adipose tissue lipolysis was inhibited in FGF21 KO mice. Intra-adipose lipolysis signaling exhibited a resistance phenotype in the KO mice, evidenced by the reduced response to ATGL and PLIN expression and p-HSL levels. However, FGF21 does not act directly on adipocytes to stimulate lipolysis. Using primary adipocytes and differentiated 3T3-L1 cells, we showed that alcohol did not increase median glycerol levels, and this lack of response was FGF21 independent. Rather, we demonstrated that the alcohol-induced catecholamine elevation is significantly attenuated in FGF21 KO mice. In addition, treatment of FGF21 KO mice with rhFGF21 significantly elevated the plasma NE. Taken together, our findings support the concept that FGF21 increases SNS activity to increase the release of catecholamine, which mediates the stimulatory effect on alcohol-induced lipolysis.

The role of FGF21 on adipose lipolysis is still elusive. While some studies suggested that FGF21 stimulates glycerol secretion from adipocytes (10), other studies showed that FGF21 was likely a negative factor in adipose lipolysis (11). Upon binding to the FGF receptors, FGF21 activates ERK by phosphorylation, which has been shown to contribute to adipose tissue lipolysis (53, 54). However, our in vitro experiment using adipocytes does not support this notion in response to alcohol. Our results unambiguously demonstrate that FGF21 promotes adipose lipolysis under conditions of chronic-binge alcohol exposure. This stimulatory effect is likely mediated by global SNS activation. Although this hypothesis is further supported by a novel finding that FGF21 can stimulate SNS activity in brown adipose tissue (55), additional experiments are needed to confirm this direct effect on SNS activation.

Multiple studies have demonstrated that FGF21 plays a critical role in hepatic lipid accumulation (9, 56). In a parallel study, we showed that lack of FGF21 exacerbated chronic alcohol exposure-induced fatty liver through SIRT-1-mediated fatty acid β-oxidation (unpublished observations). However, in the chronic-binge alcohol exposure model, hepatic fat accumulation tended to decrease in FGF21 KO mice. The discrepancy clearly indicates diverse roles of FGF21 in different alcohol exposure models. Multiple studies have shown the complexity of FGF21 in metabolic disease. The different roles of FGF21 may depend on the expression and activation of FGF receptors and the cofactor, β-klotho, involved in different forms metabolic stress (57). Accumulation of hepatic fat due to alcohol ingestion is derived from two major sources: in situ hepatic events for lipid handling, including lipogenesis and fat clearance; and adipose events characterized by lipolysis. A lack of FGF21 would increase fat synthesis and decrease the clearance in the liver, but would decrease adipose fat degradation and fatty acid reverse transport into the liver. Our findings support the concept that, in global FGF21 KO mice, in situ hepatic events are dominant for chronic alcohol-induced liver fat increase, while adipose tissue lipolysis significantly compensates for the hepatic events in the chronic-binge exposure model.

It has to be noted that although there is protection on hepatic fat accumulation, there are minimal alterations in serum ALT and AST levels in FGF21 KO mice under the condition of chronic-binge alcohol exposure. The fact that most alcoholics develop fatty liver, but do not experience hepatitis and cirrhosis, has led to a second-hit hypothesis in ALD (58). Hepatic fat accumulation, as the first hit, sensitizes the liver to the second or multiple hit(s). The increased adipose tissue lipolysis and the likely increased reverse fatty acid transport into the liver by chronic-binge alcohol exposure may serve as the first hit and sensitize the liver to the second hit, which could be excessive intake of alcohol or other environmental challenges (58).

**Fig. 8.**
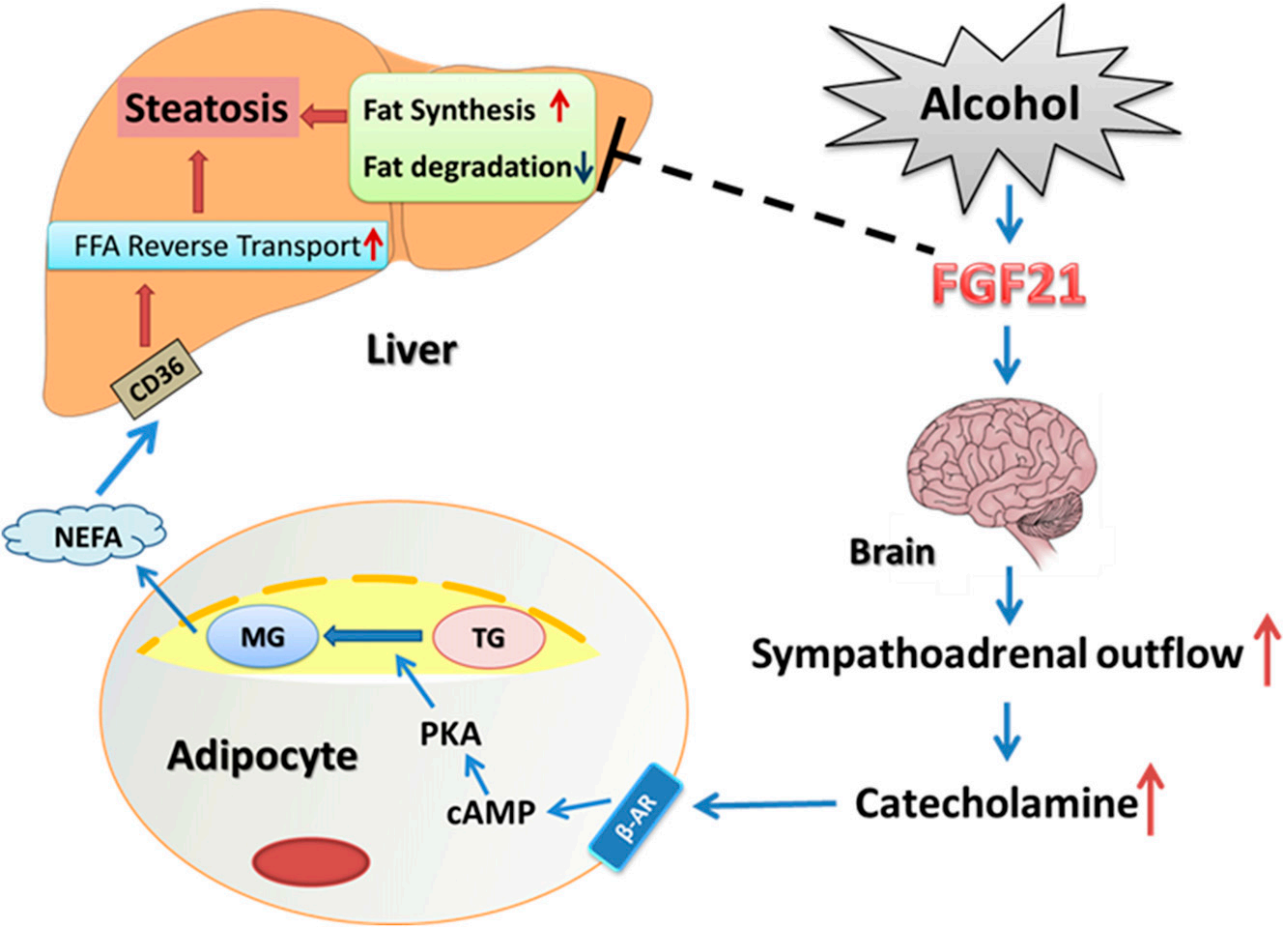
A working model of how FGF21 mediates alcohol-induced adipose tissue lipolysis by activation of the systemic release of catecholamine in mice. Alcohol exposure increases systemic FGF21 level which activates SNS to release catecholamine in the circulation. Catecholamine increases adipose tissue fat degradation by binding to β-AR leading to increased circulating NEFA concentration, which increases NEFA reverse transport into the liver and hepatic steatosis. CD36, cluster of differentiation 36; MG, monoacylglycerol.

In conclusion, as depicted in **Fig. 8**, the present study indicates that FGF21 plays a pivotal role in alcohol-induced adipose tissue lipolysis through global catecholamine regulation. Although studies have described the protective role of FGF21 in diet-induced liver lipid metabolism, its relevance to various human diseases and underlying mechanisms is not fully understood. We have demonstrated that anti-FGF21 may be beneficial for the reduction of alcohol-induced excess-lipolysis. Therefore, strategies targeting the SNS activation to block FGF21-mediated excess-adipose lipolysis might be developed to prevent/treat acute alcohol-induced fatty liver disease.

## Supplementary Material

Supplemental Data
