# Supplementary material for: FGF21 mediates alcohol-induced adipose tissue lipolysis by activation of systemic release of catecholamine in mice
Source: J Lipid Res. 2015 Aug;56(8):1481–91. doi: 10.1194/jlr.M058610 (PMC4513989; doi:10.1194/jlr.M058610)
Supplement: Supplemental Data [file supp_56_8_1481__index.html]

FGF21 Mediates Alcohol-Induced Adipose Tissue Lipolysis by Activation of Systemic Release of Catecholamine in Mice — FGF21 mediates alcohol-induced adipose tissue lipolysis by activation of systemic release of catecholamine in mice — Supplemental Data 

# FGF21 mediates alcohol-induced adipose tissue lipolysis by activation of systemic release of catecholamine in mice

## Supplemental Data

- Supplementary material - Supplementary material
