# Supplementary material for: FGF21 mediates alcohol-induced adipose tissue lipolysis by activation of systemic release of catecholamine in mice
Source: J Lipid Res. 2015 Aug;56(8):1481–91. doi: 10.1194/jlr.M058610 (PMC4513989; doi:10.1194/jlr.M058610)

SUPPLEMENTARY MATERIAL

1. Material and Methods

Blood Metabolite Assays

Mouse blood samples were centrifuged at 1500 *g* for 30 min at 4°C to obtain plasma, and stored at -80 °C for later biochemical measurements. Plasma variables were measured using commercial kits closely following the manufacturer's instructions. Alanine aminotransferase (ALT) and aspartate aminotransferase (AST) levels were measured using ALT and AST Assay kits (Thermo Fisher Scientific Inc., Middletown, VA) respectively. Free fatty acids and triglyceride levels were quantified using commercial kits (Wako Chemicals, Richmond, VA). Plasma glycerol level was quantified using a commercial kit (Cayman, Ann Arbor, Michigan, USA). Insulin was measured with Insulin Ultrasensitive ELISA kit (ALPCO Diagnostics, Salem, NH). Epinephrine (EP) and Norepinephrine (NE) were measured using Adrenaline (EP) or Noradrenaline (NE) Research ELISA kits (Labor Diagnostica Nord, Nordhorn, Germany). FGF21 concentrations in the plasma were measured using ELISA kit (Biovendor, Modrice, Czech Republic).

Isolation of primary hepatocytes and AML-12 cell culture

Primary hepatocytes were isolated from the WT and the KO mice by *in situ* digestion of the liver with perfusion of collagenase type IV. Briefly, total liver tissues were perfused with EGTA solution (10 mM HEPES [pH 7.4], 5 mM glucose, 138 mM NaCl, 5.4 mM KCl, 28.3 mM $NaHCO_3$, 0.12 mM Na_2HPO_4, 0.56 mM NaH_2PO_4 and 0.5 mM EGTA) into the inferior vena cava. After perfusion, the liver tissues were dissociated into hepatocytes using collagenase solution (10 mM HEPES [pH 7.4], 138 mM NaCl, 5.4 mM KCl, 28.3 mM $NaHCO_3$, 0.12 mM Na_2HPO_4, 0.56 mM NaH_2PO_4 supplemented with 0.0857 U/ml type IV collagenase (Roche Diagnostics, Indianapolis, IN) and 3.8 mM $CaCl_2$). Subsequently, the isolated

hepatocytes were washed with serum-free Waymouths medium (Gibco BRL, Life Technologies, Inc., Grand Island, NY) and suspended in Waymouths medium supplemented with 10% (w/v) fetal bovine serum (FBS), antibiotic-Antimycotic (Gibco, Grand Island, NY) and insulin-transferrin-selenium (ITS) supplement (Lonza, Walkersville, MD). Cell viability was assessed by the trypan blue exclusion test. Isolated hepatocytes were seeded at a density of 3.5×10^5 cells/dish in 35-mm tissue culture dishes and maintained at 37°C in 5% CO_2. After cell attachment (approximately 4 hours), the culture media were replaced with fresh media for treatment.

Mouse AML-12 hepatocytes were provided by Dr. Min You. They were cultured in DMEM/F12 medium (ATCC) supplemented with 10% FBS, 100 μg/ml streptomysin, 100 Unit/ml penicillin, 0.1 μM dexamethasone and ITS (Gibco, Grand Island, NY). Mouse primary hepatocytes and AML-12 cells were incubated with 200 mM EtOH for 4 hours, total RNA was extracted and FGF21 mRNA levels were determined.

Differentiation of 3T3-L1 adipocytes and primary adipocytes isolation

3T3-L1 adipocytes (ATCC) were cultured in Dulbecco's Modified Eagle Medium (DMEM) that contained high glucose (4.5 g/L), glutamine (584 mg/L), and sodium pyruvate (110 mg/L) supplemented with 10% newborn calf serum (NCS) and maintained at 37°C in 5% CO_2. Differentiation was induced 2 days post confluency by the addition of DMEM containing 10% FBS and dexamethasone (1 μM), 3-isobutyl-1-methylxantine (IBMX) (0.5 mM) and insulin (1 mg/L) for 2 days. The cells were further incubated with DMEM/FBS supplemented with insulin for an additional 2 days. Fully differentiated adipocytes were maintained in DMEM/FBS at 37 °C in 5% CO_2 and used 9–14 days after the initiation of differentiation.

For primary adipocytes isolation, epididymal fat pads (eWAT) from WT and FGF21 KO mice were placed in isolation buffer (pH 7.4, DMED containing 25mM HEPES, 5 mM glucose, 1% BSA (fraction

V), antibiotics (penicillin and streptomycin-100 μg/ml each) and 1mg/ml collagenase type I) and digested for 30 min at 37°C. The digestion mixture was then filtered through a nylon strainer and centrifuged at 100 *g* for 2 min. The oily layer (released from broken cells) above floating fat cells was skimmed off, and fat cells were recovered from the top, washed three times, and then resuspended in incubation buffer (pH 7.4, DMED containing 25 mM HEPES, 5 mM glucose, 1% BSA (fraction V), antibiotics (penicillin and streptomycin-100 μg/ml each) and 10% FBS).

The stimulation of lipolysis was accomplished by incubating differentiated 3T3-L1 adipocytes and primary adipocytes with 1 μM isoproterenol (ISO) with or without the addition of propranolol (50 μM) for 2 hours or treated with 200 mM EtOH or 1 μg/ml recombinant human FGF21 (rhFGF21) for 24 hours in DMEM supplemented with 5% fatty acid-free bovine serum albumin. Median glycerol content was used to measure the extent of lipolysis, Median glycerol concentration was quantified using commercial kit (Cayman, Ann Arbor, Michigan, USA).

Liver triglyceride assay

For the liver triglyceride assay, 70–100 mg of liver tissue was homogenized in 1 ml of 50 mM NaCl. Homogenate (500 μl) was mixed with 4 ml of the extraction reagent (methanol: chloroform = 1:2) and incubated overnight at 4°C before being centrifuged at 1,800 *g* for 20 min at room temperature. The lower chloroform phase was carefully collected and dried using a speed vac, and the pellets were used for triglyceride assay using the Triglyceride Kit (Thermo Fisher Scientific Inc).

Immunohistochemistry

The liver and adipose tissue sections were fixed in formalin and embedded in paraffin. The sliced liver and adipose tissue sections were then stained with H&E as described previously(14).

Real time Quantitative RT-PCR

The mRNA levels were assessed by real-time RT-PCR. In brief, total RNA was isolated using Trizol according to manufacturer's protocol (Invitrogen, Carlsbad, CA) and reverse-transcribed using GenAmp RNA PCR kit (Applied Biosystems, Foster City, CA). The cDNA was amplified in 96-well reaction plates with a SYBR green PCR Master Mix (Applied Biosystems) on an ABI 7500 real-time PCR thermocycler. The sequences of forward and reverse primers are listed in Supplementary Table 1. The relative quantities of target transcripts were calculated from duplicate samples after normalization by a housekeeping gene, 18s. Dissociation curve analysis was performed after PCR amplification to confirm the specificity of the primers. Relative mRNA expression was calculated using the $^{\Delta\Delta}$Ct method.

Western blot analysis

Western blot was performed as described previously to detect p-AKT, AKT, p-HSL, HSL, ATGL, PLIN, p-ERK, ERK, p-(Ser/Thr) PKA Substrate (Cell Signaling Technologies), FGF21 (Abcam, San Francisco, CA), β-actin (Santa Cruz Biotechnology). Blots were scanned using a Bio-Rad Imaging System (Image Lab™ Upgrade for ChemiDoc™ XRS+ System #170-8299). All specific bands were quantified with the Automated Digitizing System (Image Lab 4.1 programs). Results are representative of three independent experiments.

Cyclic AMP (cAMP) concentration

cAMP levels were measured in epididymal white adipose tissue (eWAT) using cAMP direct immunoassay kit (Abcam, Cambridge, MA) according to the manufacturer's protocol.

2. Supplementary Table

Supplementary Table 1. Primer Sequences for Real-Time Quantitative RT-PCR analysis

3. Supplementary Figures

Supplementary Figure 1. Animal feeding schedule. Mice were initially fed the Lieber-DeCarli diet containing 1.6% (vol/vol) alcohol for 3 days and 3.3% (vol/vol) alcohol for another 3 day to acclimatize them to liquid diet and tube feeding. Afterward, the alcohol (EtOH)-fed group were allowed free access to Lieber-DeCarli diet containing 5 % (vol/vol) alcohol for 12 days. Mice in control groups were pair-fed with isocaloric control diet. On the last day of experiment, AF and PF mice were gavaged in the early morning with a single dose of alcohol (5g/Kg body weight) or isocaloric maltose dextrin, respectively. Mice were killed 6 hours later. One group of alcohol-exposed FGF21 KO mice were treated with 4 mg/Kg rhFGF21(KO+AF+rhFGF21) daily via intraperitoneal injection during the last 5 days.

Supplementary Figure 2. The Adipocyte does not respond to FGF21 and alcohol to induce lipolysis. (A) Fully differentiated 3T3-L1 adipocytes were incubated with 1μg/ml rhFGF21 for 5 minutes. Cell lysates were immunoblotted for phosphor-p42/44- or total ERK as indicated. (B) Primary adipocytes isolated from epididymal adipose tissue in WT and FGF21 KO mice were incubated with alcohol (EtOH, 200mM, 24h) or rhFGF21 (1 μg/ml, 24h), isoproterenol (ISO,1μM) with or without propranolol (50 μM) was added to the culture and incubated for 2 hours. Median glycerol levels were determined. (C) Fully differentiated 3T3-L1 adipocytes were treated as descried in Supplementary Figure 2B, median glycerol levels were determined. Data are expressed as mean ± SEM. (*, $P<0.05$)

Supplementary Figure 3. Expression of β-adrenergic receptors (AR) in eWAT after chronic-binge alcohol feeding. Mice were fed as descried in Figure 1. (A) Expression of three β-AR isoforms expressed as $^{\Delta}$Ct values (mean β-AR Ct- 18s Ct). (B) Changes in β1-AR expression in eWAT (AF/PF). (C)

Changes in β2-AR expression in eWAT (AF/PF). (D) Changes in β3-AR expression in eWAT (AF/PF). N=7-14 mice per group.

Supplementary Table 1. Primer Sequences for Real-Time Quantitative RT-PCR analysis

**Genes**	**Forward primers (5’-3’)**	**Reverse primers (5’-3’)**
PNMT	GACTGGAGTGTGTATAGTCAGCA	CGATAGGCAGGACTCGCTTC
β1-AR	TGGCTTACTGGCTTGTCTTG	TTTCCACTCGGGTCCTTG
β2-AR	GGACAACCTCATCCCTAA	AGAGTAGCCGTTCCCATA
β3-AR	CAGTCCCTGCCTATGTTTG	TTCCTGGATTCCTGCTCT
FGF21	CCTCTAGGTTTCTTTGCCAACAG	AAGCTGCAGGCCTCAGGAT
18S	CATTCGAACGTCTGCCCTAT	CCTGCTGCCTTCCTTGGA

3. Supplementary Figures

Supplementary Figure 1. Animal feeding schedule. Mice were initially fed the Lieber-DeCarli diet containing 1.6% (vol/vol) alcohol for 3 days and 3.3% (vol/vol) alcohol for another 3 day to acclimatize them to liquid diet and tube feeding. Afterward, the alcohol (EtOH)-fed group were allowed free access to Lieber-DeCarli diet containing 5 % (vol/vol) alcohol for 12 days. Mice in control groups were pair-fed with isocaloric control diet. On the last day of experiment, AF and PF mice were gavaged in the early morning with a single dose of alcohol (5g/Kg body weight) or isocaloric maltose dextrin, respectively. Mice were killed 6 hours later. One group of alcohol-exposed FGF21 KO mice were treated with 4 mg/Kg rhFGF21(KO+AF+rhFGF21) daily via intraperitoneal injection during the last 5 days.

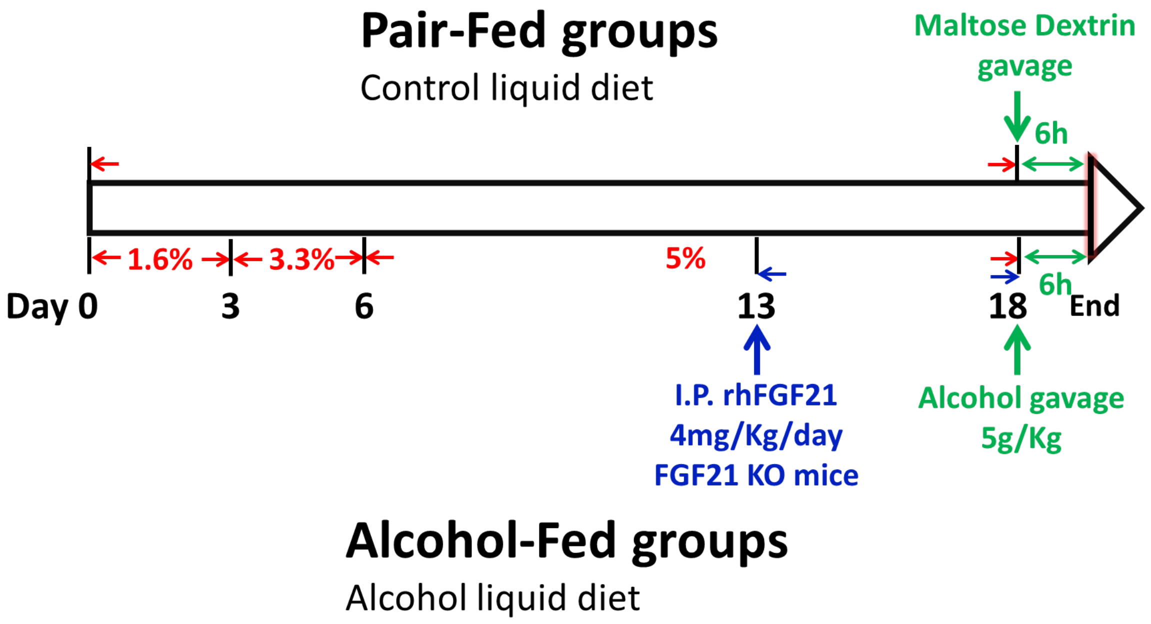

Supplementary Figure 2. The Adipocyte does not respond to FGF21 and alcohol to induce lipolysis. (A) Fully differentiated 3T3-L1 adipocytes were incubated with 1µg/ml rhFGF21 for 5 minutes. Cell lysates were immunoblotted for phosphor-p42/44- or total ERK as indicated. (B) Primary adipocytes isolated from epididymal adipose tissue in WT and FGF21 KO mice were incubated with alcohol (EtOH, 200mM, 24h) or rhFGF21 (1 µg/ml, 24h), isoproterenol (ISO,1µM) with or without propranolol (50 µM) was added to the culture and incubated for 2 hours. Median glycerol levels were determined. (C) Fully differentiated 3T3-L1 adipocytes were treated as descried in Supplementary Figure 2B, median glycerol levels were determined. Data are expressed as mean ± SEM. (*, P<0.05)

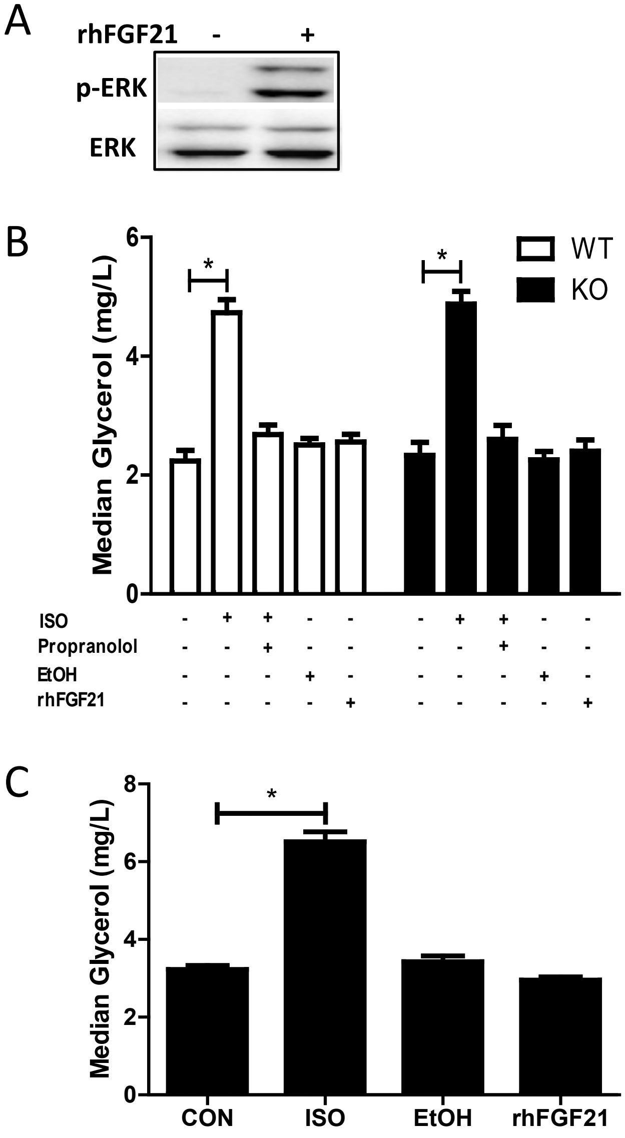

Supplementary Figure 3. Expression of β-adrenergic receptors (AR) in eWAT after chronic-binge alcohol feeding. Mice were fed as descried in Figure 1. (A) Expression of three β-AR isoforms expressed as $^{\Delta}$Ct values (mean β-AR Ct- 18s Ct). (B) Changes in β1-AR expression in eWAT (AF/PF). (C) Changes in β2-AR expression in eWAT (AF/PF). (D) Changes in β3-AR expression in eWAT (AF/PF). N=7-14 mice per group.

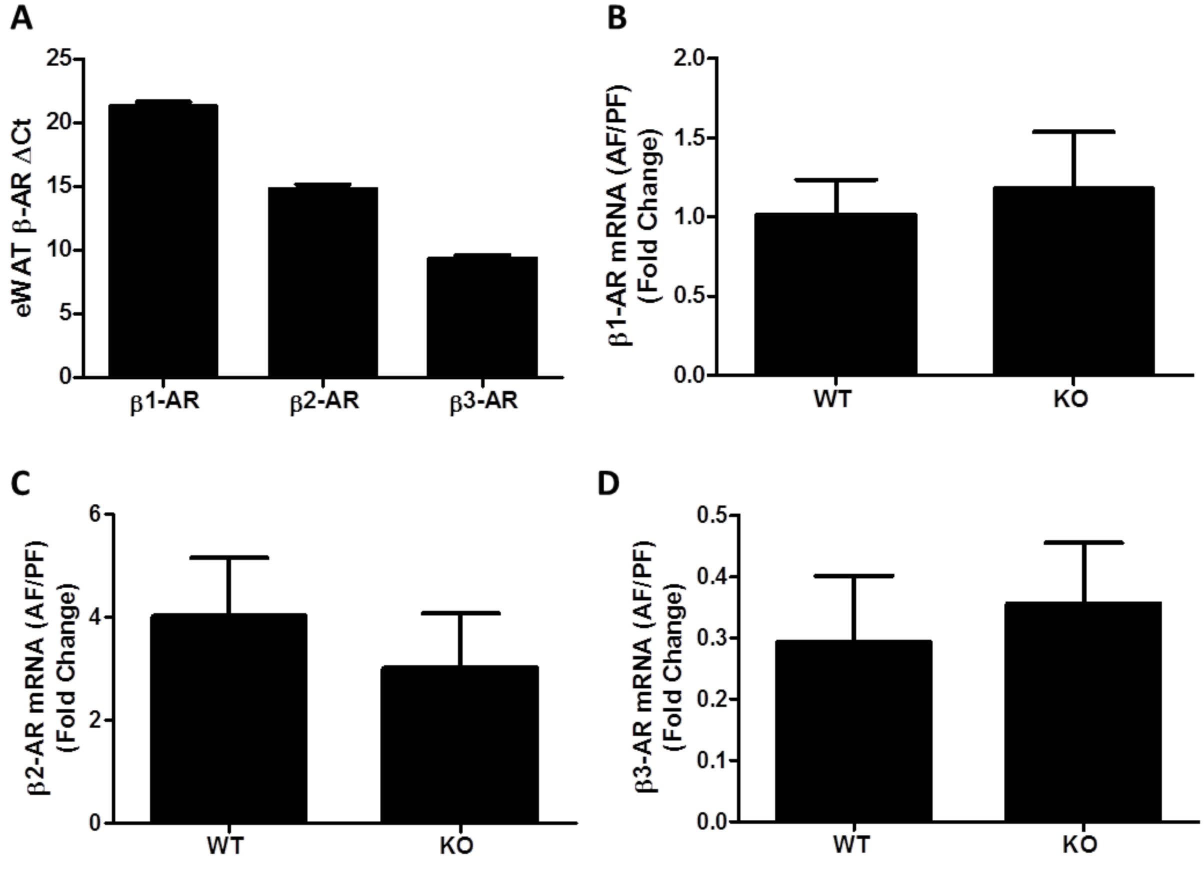

Supplement: Supplemental Data [file supp_M058610_jlr.M058610-1.pdf]
